# The Antioxidant Supplementation with *Filipendula ulmaria* Extract Attenuates the Systemic Adverse Effects of Nanosized Calcium Phosphates in Rats

**DOI:** 10.1155/2021/8207283

**Published:** 2021-08-17

**Authors:** Radomir Scepanovic, Dragica Selakovic, Jelena S. Katanic Stankovic, Natalija Arsenijevic, Marija Andjelkovic, Jovana Milenkovic, Pavle Milanovic, Miroslav Vasovic, Nemanja Jovicic, Gvozden Rosic

**Affiliations:** ^1^Military Medical Academy, University of Defense, Belgrade, Serbia; ^2^Department of Physiology, Faculty of Medical Sciences, University of Kragujevac, 34000 Kragujevac, Serbia; ^3^Department of Science, Institute for Information Technologies Kragujevac, University of Kragujevac, 34000 Kragujevac, Serbia; ^4^Department of Dentistry, Faculty of Medical Sciences, University of Kragujevac, 34000 Kragujevac, Serbia; ^5^Department of Biochemistry, Faculty of Medical Sciences, University of Kragujevac, Kragujevac, Serbia; ^6^Department of Histology and Embryology, Faculty of Medical Sciences, University of Kragujevac, 34000 Kragujevac, Serbia

## Abstract

The aim of this study was to investigate and compare the systemic toxicity of three nanosized calcium phosphates (CaPs): hydroxyapatite (HA), tricalcium phosphate (TCP), and amorphous calcium phosphate (ACP) in rats. Since those metallic compounds are widely used as bone replacement materials, including their use in oral surgery, CaPs were applied (*per os*) equimollary (17.8 mg/kg, 11 mg/kg, and 9.65 mg/kg b.w., respectively) for 30 days in order to mimic the previously described release rate from dental composites. Also, we employed antioxidant supplementation with *Filipendula ulmaria* (FU) extract. All the applied CaPs significantly increased serum calcium, triglycerides, LDL, and LDH, while serum levels of testosterone and LH declined, with no alterations in the liver enzymes. The evaluation of oxidative stress markers (in the liver, kidney, and testicle) showed an increase in TBARS values, while SOD and CAT activities and GSH levels were significantly reduced. The relative gene expression of Bax and Bcl-2 was shifted to proapoptotic action, accompanied by intense characteristic histological changes in architecture in all investigated organs. The toxic effects were most prominent in groups treated by ACP. FU administration attenuated the majority of nanosized CaP-induced adverse effects, thus recommending this therapeutic approach to minimize nano-CaP systemic toxicities.

## 1. Introduction

Calcium phosphates (CaPs) represent compounds of special interest in many scientific fields, with the evidently growing need for their use in medicine. In contrast to the synthetic polymers used in biomedical applications, those metal compounds are naturally present in the human body [1]. Due to the fact that CaPs express the highest levels of biocompatibility, it is not surprising that they are well accepted by the body and that they are optimally integrated into a human body [2]. They are widely accepted as materials of choice for hard tissue regeneration [3], artificial bone substitution [4], and treatment of bone defects [5]. However, the most frequent application of CaPs usually takes place in various interventions in dentistry, such as numerous endodontic treatments [6], coating in dental implants [7], failing subperiosteal metal implants [8], periodontal defects [9], and restoration of edentulous atrophic ridges [10]. From the clinical point of view, numerous investigations have been conducted with different CaP polymers, including hydroxyapatite (HA), monocalcium phosphate, dicalcium phosphate, tricalcium phosphate (TCP), tetracalcium phosphate, and amorphous calcium phosphate (ACP), as bioactive components of composites [11]. However, recent advances in biomaterials direct clinical indications for CaP application to nanoparticles in order to increase their remineralization potential [12].

Nevertheless, despite a number of studies that lead to a conclusion for the absolute safety of those composites, it has been reported that the medical administration of CaPs is accompanied by growing concerns based on their adverse effects. Even though there are only a few published reports evaluating nano-CaP toxicities, it is possible to notice that the finding is evidently in accordance with their results. Namely, it has been confirmed that nano-CaP (HA, TCP, and ACP) administration is accompanied by various systemic morphofunctional abnormalities. In both the *in vitro* and *in vivo* investigations, nano-CaP application resulted in increased oxidative stress [13], apoptosis [14], and DNA damage [15], which were accompanied by various structural alterations. Moreover, numerous tissues and organs were affected by nano-CaPs. Depending on the investigated organ system, there are confirmations for neurotoxicity [11], nephrotoxicity [16], hepatotoxicity [17], and gonadotoxicity [18]. Since the common pathophysiological mechanisms of nano-CaP toxicities involve oxidative damage, with consequent apoptotic turnover, it is not surprising that cosupplement application of antioxidants has become a routine procedure in the treatment of nano-CaP-induced toxicities. At the same time, the use of natural products, as compounds abundant in the antioxidant defense potential, is currently a topic of actual interest for medical applications. Therefore, based on the results of several reports, it seems that toxicities induced by different CaPs could be attenuated by a variety of antioxidant-rich products.

*Filipendula ulmaria* (L.) Maxim. (Rosaceae) (FU) is a medicinal plant widely investigated for its confirmed antioxidant, antimicrobial, anti-inflammatory, and antiproliferative properties [19–23]. In the aerial part of the plant, there is considerable content of phenolic compounds, especially total phenolic acids and flavonoids [21]. It has been described that FU extract exerted high antioxidant activities [24]. In addition, our recent investigation confirmed a beneficial response to FU in the prevention of behavioral manifestations in nano-CaP-induced neurotoxicity [11].

Due to the evident lack of comparative information on the toxicity of those nanoparticles, the aim of this study was to evaluate the potential systemic effects of different CaPs applied in the form of nanoparticles, by means of the morphofunctional status of the kidney, liver, and testis. Furthermore, following the possible involvement of oxidative stress in the mediation of nano-CaP systemic actions, we included the simultaneous administration of antioxidant-rich *Filipendula ulmaria* extract, as a potential therapeutic approach that could minimize the role of oxidative stress in the pathogenesis of nano-CaP systemic toxicities.

## 2. Materials and Methods

### 2.1. Animals and Treatment

**Figure 1:**
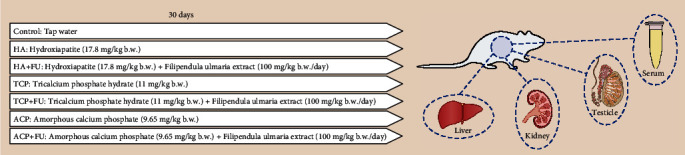
Experimental design.

A total of 42 male Wistar albino rats (2 months old, 250–300 g) that were used in the study were purchased from the Military Medical Academy (Serbia) and housed in groups of three per transparent polyethylene cage under standard environmental conditions (temperature: 23 ± 1°C; light: 12/12 h light/dark cycle). The rats had access to food and water *ad libitum*. The animals were randomly divided into seven equal groups as follows: control group; three groups that orally (dissolved in tap water) received individual nanosized calcium phosphates: hydroxyapatite (17.8 mg/kg b.w.) (HA) group, tricalcium phosphate hydrate (11 mg/kg b.w.) (TCP) group, and amorphous calcium phosphate (9.65 mg/kg b.w.) (ACP) group; and three groups that along with calcium phosphates simultaneously orally received *Filipendula ulmaria* extract (100 mg/kg b.w.): HA+FU, TCP+FU, and ACP+FU groups (Figure 1).

The mineral components of dental composites in nanoparticles were purchased from Sigma-Aldrich, Germany: hydroxyapatite nanopowder: <200 nm particle size (BET), ≥97%, and synthetic; tricalcium phosphate hydrate nanopowder: <200 nm particle size (BET); and calcium phosphate: amorphous nanopowder and <150 nm particle size (BET). The doses for nanosized calcium phosphates were determined to meet the following criteria, as previously described by Arsenijevic and coworkers [11]: to achieve the equimolarity, to level the lowest dose of calcium phosphates (HA) that showed toxic effects according to literature data [25] for experiments performed *in vivo* with nanosized particles, and to achieve the doses of calcium phosphates daily released from dental composites *in vitro* [26]. The route of administration (*per os*) was chosen to mimic the authentic way of application in humans. From the plant material (aerial part of FU), the preparation of the extract, as well as the content identification, was performed by using the previously described procedure [24]. The daily dose of FU extract was selected according to the results of our previous study with confirmed biological effectiveness of this extract [24]. The final concentrations of all substances applied in this study were determined according to the average water intake measured in the past 24 hours. All applied protocols lasted for 30 days, with continuous monitoring by a veterinarian on a daily basis. The day before sacrifice, food intake was totally restricted to all animals at 8 pm.

### 2.2. Sample Preparation

Twenty-four hours after completing the experimental protocols, the animals were anesthetized by short-term narcosis, induced by intraperitoneal application of ketamine (10 mg/kg) and xylazine (5 mg/kg), and then sacrificed by decapitation. Trunk blood samples were collected for the determination of serum biochemical parameters and sex hormone levels, while (simultaneously) organs (kidney, liver, and testis) were rapidly removed for tissue sample analysis. The collected blood samples were allowed to clot at room temperature for 2 h in anticoagulant-free tubes and then centrifuged at 3500 g for 15 min at 4°C. The clear supernatant was kept at −80°C until analysis. The tissue supernatants were obtained from the fresh liver, kidney, and testis tissues using phosphate-buffered saline (PBS; 0.01 M, pH 7.4). Briefly, fresh tissue was homogenized with PBS in a 1 : 5 ratio using a manual tissue homogenizer and centrifuged at 4000 rpm for 15 min at 4°C. Obtained supernatants were carefully separated and stored in the freezer until further use.

### 2.3. Biochemical Parameter Determination

The biochemical parameters—creatinine, urea, calcium, triglycerides, total cholesterol, HDL (high-density lipoprotein) cholesterol, LDL (low-density lipoprotein) cholesterol, lactate dehydrogenase (LDH), alkaline phosphatase (ALP), alanine aminotransferase (ALT), and aspartate aminotransferase (AST)—were estimated by using standard kits in an automatic clinical chemistry analyzer (Abbott Alinity c analytical system, Abbott Core Laboratory, Abbott Diagnostics).

### 2.4. Serum Hormone Assays

The testosterone and LH (luteinizing hormone) levels were measured by the enzyme-linked immunosorbent assay kits, according to the manufacturer's instructions (Abcam, Cambridge, MA, USA). The limits of detection were 6 pg/mL and 0.01 ng/mL, respectively. The intra- and interassay coefficients of variation were 4.5% for testosterone and 3.9% for LH.

### 2.5. The Determination of Oxidative Stress Markers

The activity of enzymes included in the protection of the organism against induced oxidative stress, catalase (CAT) and superoxide dismutase (SOD), was estimated using spectrophotometric methods reported by Beers and Sizer [27] and Misra and Fridovich [28], respectively. The level of enzymatic activity was expressed as units per milligram of proteins (U/mg) in the sample. Ellman's spectrophotometric assay [29], employing 5,5-dithio-bis-(2-nitrobenzoic acid), was used for the evaluation of the content of reduced glutathione (GSH), a significant endogenous antioxidant. The GSH content was expressed as mg GSH/g proteins. The production of thiobarbituric acid reactive substances (TBARS) during oxidative stress-induced lipid oxidation in tissues was monitored according to the method of Ohkawa and coworkers [30]. The TBARS levels were estimated as nmol of malondialdehyde (MDA)/mg proteins. The concentration of proteins in each tissue homogenate sample was evaluated by Lowry et al.'s assay [31] using bovine serum albumin as a standard. The instrument used for all measurements was a UV-Vis double-beam spectrophotometer (model Halo DB-20S, with a temperature controller, Dynamica GmbH, Dietikon, Switzerland). The preparation of tissue homogenates was done following the procedure from Kumburovic and colleagues [32].

### 2.6. Chemicals for the Oxidative Stress Determination

2-Thiobarbituric acid (TBA) was obtained from abcr GmbH (Karlsruhe, Germany). 1,1,3,3-Tetraethoxypropane (malonaldehyde bis(diethyl acetal), MDA) was purchased from Acros Organics (Geel, Belgium). Bovine serum albumin was obtained from HUMAN GmbH (Wiesbaden, Germany). All other reagents for the determination of oxidative stress parameters in tissue homogenates were purchased from Sigma-Aldrich Chemie GmbH (Darmstadt, Germany).

### 2.7. RNA Isolation and Real-Time PCR Analysis

Total RNA was extracted from the liver, renal, and testicular tissues using a PureZOL reagent (Bio-Rad, USA) according to the manufacturer's instructions. Reverse transcription was done using iScript Reverse Transcription Mastermix (Bio-Rad, USA). Quantitative RT-PCR was performed using SsoAdvanced Universal SYBR Green Supermix (Bio-Rad, USA). mRNA-specific primers for Bax, Bcl-2, and *β*-actin as a housekeeping gene were used. Quantitative RT-PCR reactions were done in the Applied Biosystems 7500 (Applied Biosystems, USA), and after data analysis, relative gene expression was calculated according to Livak and Schmittgen [33].

### 2.8. Histological Analysis

Livers, kidneys, and testes were excised, fixed in 10% buffered formalin, and embedded in paraffin. Tissue sections (5 *μ*m) were stained with H&E. Oil Red O staining was performed on liver tissue cryosections (5 *μ*m). Tissue sections were fixed in paraformaldehyde (10%), rinsed with 60% isopropanol, and stained with freshly prepared Oil Red O working solution for 10 minutes. After rinsing with 60% isopropanol, the sections were counterstained with Mayer's hematoxylin and mounted with glycerin jelly.

All research procedures were carried out in accordance with the European Directive for the welfare of laboratory animals No 86/609/EEC and the principles of Good Laboratory Practice and according to ARRIVE guidelines. All experiments were approved by the Ethical Committee of the Faculty of Medical Sciences, University of Kragujevac, Serbia.

### 2.9. Statistical Analysis

Statistical analysis was performed with the SPSS version 20.0 statistical package (IBM SPSS Statistics 20). The results are expressed as the mean ± standard error of the mean (SEM). The parameters were initially submitted to the Levene test for homogeneity of variance and to the Shapiro-Wilk test for normality. One-way ANOVA, followed by the Bonferroni post hoc test, was used for comparisons between the groups. The significance was determined at *p* < 0.05 for all tests.

## 3. Results

**Figure 2:**
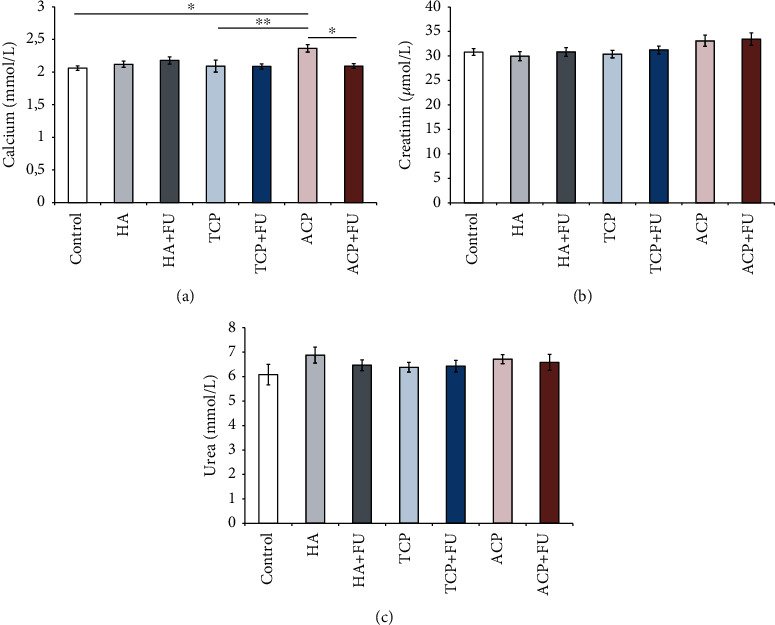
Serum levels of calcium, creatinine, and urea (a–c). The values are mean ± SEM; ∗ denotes a significant difference, *p* < 0.05; ∗∗ denotes a significant difference, *p* < 0.01.

As shown in Figure 2(a), prolonged intake of nanosized CaPs significantly altered serum calcium levels (*F* = 3.517, df = 6). The highest calcemia was observed in the ACP group, where the increase was significant when compared to the control (*p* < 0.05) and TCP groups (*p* < 0.01). However, simultaneous antioxidant supplementation with FU extract reversed ACP-induced hypercalcemia (*p* < 0.05) to the control values. In contrast, none of the applied protocols significantly influenced serum levels of creatinine (Figure 2(b), *F* = 2.002) and urea (Figure 2(c), *F* = 0.810).

**Figure 3:**
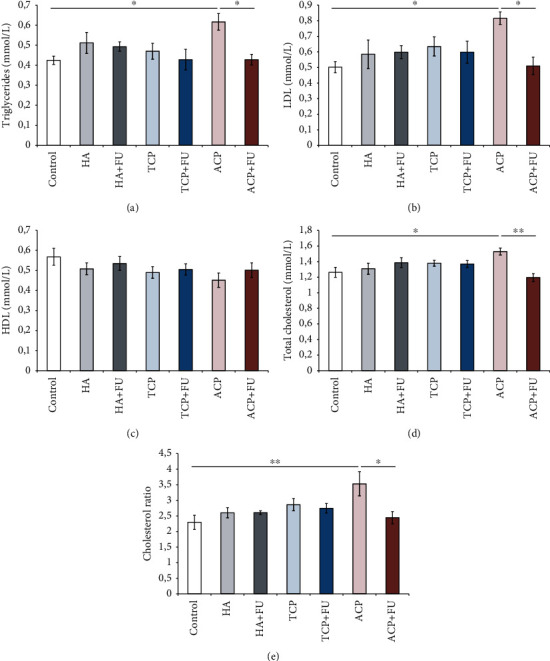
Serum levels of triglycerides, LDL, HDL, and total cholesterol and the cholesterol ratio (a–e). The values are mean ± SEM; ∗ denotes a significant difference, *p* < 0.05; ∗∗ denotes a significant difference, *p* < 0.01.

Serum markers of lipid status were significantly affected by prolonged oral administration of nanosized calcium phosphates (Figure 3). As presented in Figures 3(a) and 3(b), serum triglycerides and LDL cholesterol levels were significantly above the control values (*p* < 0.05, *F* = 3.192 and 3.086) in rats solely treated with ACP but lowered after FU extract administration (*p* < 0.05) and leveled to the control values. At the same time, serum HDL levels were not significantly affected by any of the applied nanosized CaPs (Figure 3(c), *F* = 1.139). More significant impact following the 30-day protocol with nanosized CaPs was achieved on total cholesterol levels (Figure 3(d), *F* = 3.693), where the treatment with ACP resulted in a significant increase when compared to the control (*p* < 0.05), and attenuated by FU extract administration that significantly reduced serum total cholesterol levels (*p* < 0.01) when compared with the group where only ACP was administered. The most prominent alterations of lipid status induced by ACP intake were confirmed by the cholesterol ratio (Figure 3(e), *F* = 3.386) that was also significantly increased following the treatment with nanosized ACP particles (*p* < 0.01) and declined after simultaneous FU extract administration (*p* < 0.05).

**Figure 4:**
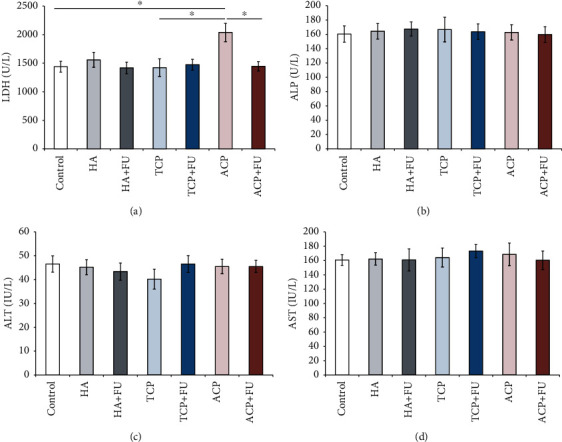
Serum levels of LDH, ALP, ALT, and AST (a–d). The values are mean ± SEM; ∗ denotes a significant difference, *p* < 0.05; ∗∗ denotes a significant difference, *p* < 0.01.

Serum LDH levels (Figure 4(a)) were seriously affected by the applied protocols (*F* = 3.457), which was manifested in the ACP group by LDH elevation when compared to both the control and TCP groups (*p* < 0.05), and reversed to the control values after FU extract application. Unlike LDH, neither of the evaluated liver enzymes, ALP, ALT, and AST (Figures 4(b)–4(d)), was significantly altered following any of the applied protocols (*F* = 0.059, 0.443, and 0.159, respectively).

**Figure 5:**
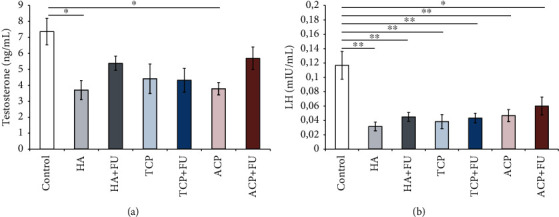
Serum levels of testosterone and LH (a, b). The values are mean ± SEM; ∗ denotes a significant difference, *p* < 0.05; ∗∗ denotes a significant difference, *p* < 0.01.

As shown in Figure 5(a), serum testosterone levels were significantly influenced by the protocols with nanosized CaPs (*F* = 3.579). Although all applied CaPs declined testosterone levels (significant for HA and ACP, *p* < 0.05), when applied along with CaP nanoparticles, FU extract prevented the lowering of the levels of testosterone. Even more significant hormonal disturbance following prolonged nanosized CaP intake was observed by means of serum LH levels (Figure 5(b), *F* = 7.130). All three applied forms of nano-CaPs induced a significant reduction in serum LH (*p* < 0.01). The deterioration of LH levels was persistent even following antioxidant supplementation with FU extract, with values significantly below the control (*p* < 0.01).

**Figure 6:**
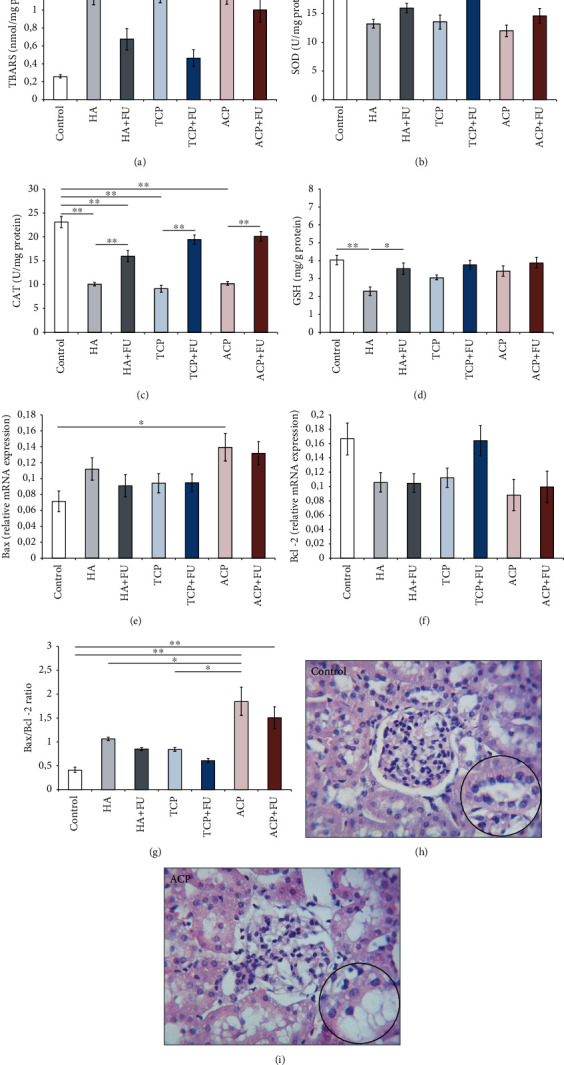
Oxidative stress (a–d) and apoptotic (e–g) markers with representative H&E staining in the kidney (h, i). The values are mean ± SEM; ∗ denotes a significant difference, *p* < 0.05; ∗∗ denotes a significant difference, *p* < 0.01.

Lipid peroxidation in kidney tissue, expressed as TBARS (Figure 6(a)), was significantly altered following the applied protocols (*F* = 12.801). TBARS values were significantly above the control values (*p* < 0.01) in all three groups solely treated with CaPs. However, the significant reduction of the lipid peroxidation was observed after simultaneous administration of FU extract in groups treated with HA and TCP, while antioxidant supplementation failed to prevent the increase in TBARS values with ACP, since the values remained significantly above the control (*p* < 0.01). The enzymatic antioxidant defense (Figures 6(b) and 6(c)) was significantly affected by the applied protocols (*F* = 7.687 and 39.195, respectively). The activity of SOD was significantly reduced by all three nano-CaPs individually (*p* < 0.05 for HA and TCP, and *p* < 0.01 for ACP) and reestablished by simultaneous antioxidant supplementation. The significant reduction of CAT activity, also achieved with all three nano-CaPs (*p* < 0.01), although significantly enhanced by FU extract (*p* < 0.01), still remained significantly below the control values (*p* < 0.01) in all three combined groups. Nonenzymatic antioxidant capacity (Figure 6(d)), like the enzymatic mechanism, was also significantly influenced by the applied treatments (*F* = 5.248). The significant reduction of GSH levels was observed following HA administration (*p* < 0.01) but was successfully compensated by FU extract that resulted in a significant increase of kidney tissue GSH concentration (*p* < 0.05). The impact of nanosized CaPs on the relative expression of apoptosis-related genes was estimated by the determination of the Bax, Bcl-2, and Bax/Bcl-2 ratio (Figures 6(e)–6(g), *F* = 3.081, 2.919, and 12.099, respectively). While the relative mRNA expression of Bax was significantly enhanced only in the ACP group (*p* < 0.05) and successfully attenuated by FU extract administration, no significant impact of the applied protocols was observed in antiapoptotic Bcl-2. However, when expressed as a pro/antiapoptotic gene expression ratio, the nanosized CaPs significantly shifted the ratio to proapoptotic. Although this ratio was observed with all three nano-CaPs, the significant augmentation was achieved only with ACP. ACP administration resulted in the Bax/Bcl-2 ratio enhancement even when compared to the other two CaPs used in this study (*p* < 0.05), and it was persistently low following antioxidant supplementation with FU extract. Observed results were in line with pathohistological findings of vacuolation of the proximal convoluted tubule epithelium and glomerular cells that were most prominent in the ACP group (Figures 6(h) and 6(i)).

**Figure 7:**
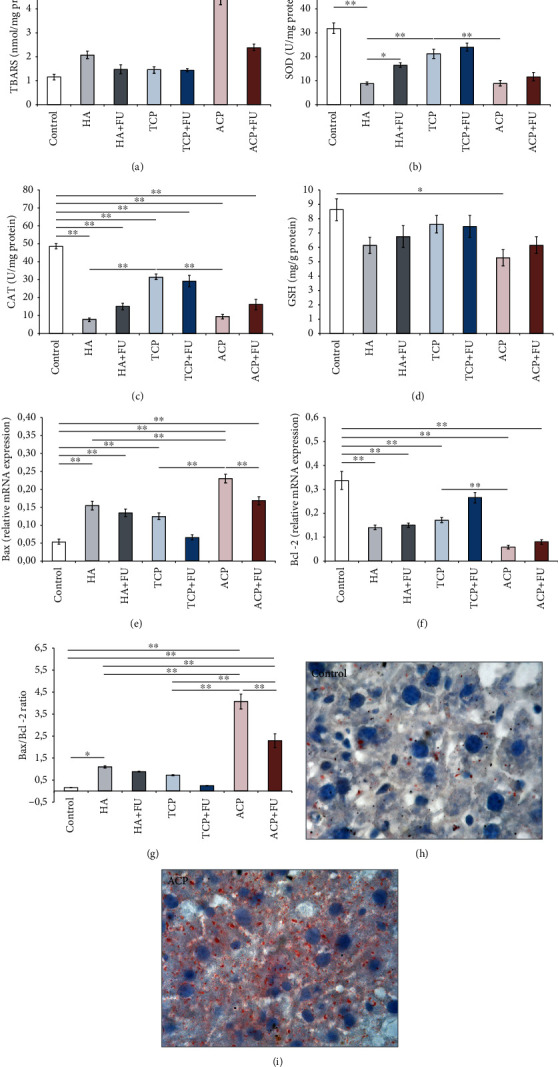
Oxidative stress (a–d) and apoptotic (e–g) markers with representative photomicrographs of Oil Red O staining in the liver (h, i). The values are mean ± SEM; ∗ denotes a significant difference, *p* < 0.05; ∗∗ denotes a significant difference, *p* < 0.01.

As presented in Figure 7(a), the applied protocols with nanosized CaPs showed a significant impact on lipid peroxidation, expressed as TBARS (*F* = 40.750), in the liver. While TCP administration did not produce a significant alteration of TBARS, oral intake of HA induced a significant increase when compared to the control values (*p* < 0.05). However, the most prominent enhancement of lipid peroxidation was observed following ACP application. The TBARS values in animals solely treated with ACP were significantly above the control values (*p* < 0.01), even when compared to the HA and TCP groups (*p* < 0.01). Although supplementation with FU extract significantly decreased lipid peroxidation induced by ACP (*p* < 0.01), the values remained significantly above the control (*p* < 0.01). The activity of antioxidant enzymes (Figures 7(b) and 7(c)) was also significantly altered by the applied protocols (*F* = 29.663 and 51.663, respectively). All three applied nanosized CaPs significantly reduced the activity of both SOD and CAT when compared to the control values (*p* < 0.01). Interestingly, the decline in SOD and CAT activities observed in the HA and ACP groups was even more pronounced when compared to that in the TCP group (*p* < 0.01). Although antioxidant supplementation resulted in augmentation of enzymatic activity (significant for SOD in the HA+FU group, *p* < 0.05), the values in all combined groups remained significantly below the control (*p* < 0.01). The nonenzymatic antioxidant capacity in the liver (Figure 7(d)) was less affected than the enzymatic one (*F* = 2.889), since only individual administration of ACP resulted in a significant decline (*p* < 0.05), which was also reversed by supplementation with FU extract. The analysis of the relative gene expression of apoptotic markers revealed a very strong influence of nanosized CaP administration on both the proapoptotic (Figure 7(e), *F* = 34.431) and antiapoptotic (Figure 7(f), *F* = 29.141) markers, as well as on their ratio (Figure 7(g), *F* = 62.482). When individually applied, all three CaPs employed in this study significantly enhanced the relative expression of Bax (*p* < 0.01), with the most prominent proapoptotic action observed in the ACP group, where the values were also significantly above the HA and TCP groups (*p* < 0.01). The antioxidant supplementation with FU extract, which significantly lowered Bax relative expression in the ACP combined group (*p* < 0.01), was insufficient to prevent the nano-CaP-induced increase of Bax, except in the TCP+FU group. In the same manner, the trials with individually applied nanosized CaPs significantly declined Bcl-2 relative gene expression when compared to the control (*p* < 0.01). Again, the most prominent alteration was observed in the ACP group, where the deterioration of Bcl-2 expression was significant even when compared to the TCP group (*p* < 0.01). Like Bax, the antioxidant supplementation successfully prevented the decline of this antiapoptotic marker only in the TCP+FU group. Finally, the Bax/Bcl-2 ratio confirmed the proapoptotic effect of the protocols with the individual nanosized CaPs when compared to the control (*p* < 0.05 for HA, and *p* < 0.01 for ACP), except in the TCP group. The proapoptotic action was the most prominent in the ACP group again, with the values of the Bax/Bcl-2 ratio significantly above the other two CaPs (*p* < 0.01). Unlike HA and TCP, simultaneous administration of FU extract in the applied dose, although it significantly reduced the ratio (*p* < 0.01), failed to prevent ACP-induced proapoptotic action (*p* < 0.01). Pathohistological analysis of accumulated lipids in liver tissue sections demonstrated intralobular microvesicular steatosis, which was the most prominent in the ACP group (Figures 7(h) and 7(i)).

**Figure 8:**
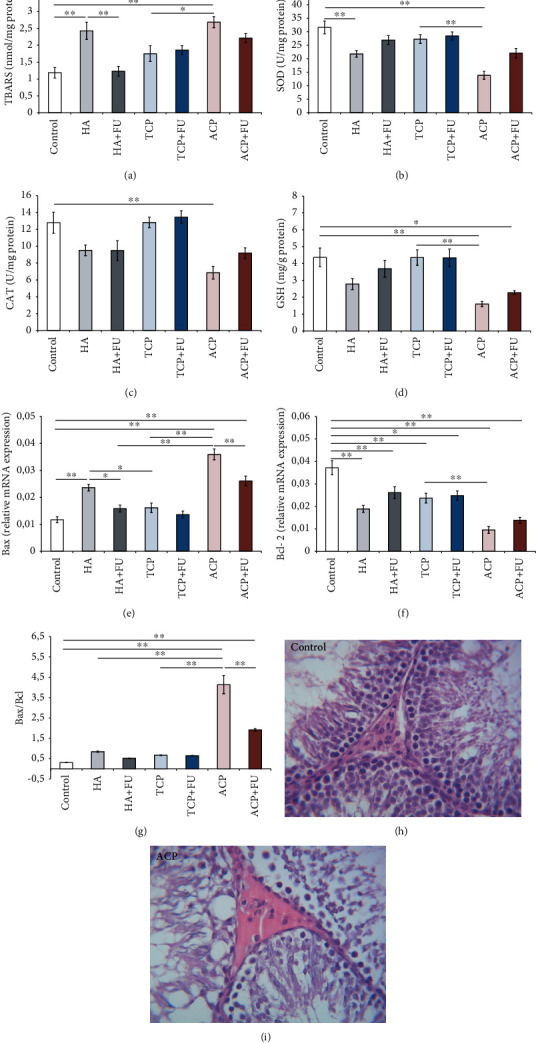
Oxidative stress (a–d) and apoptotic (e–g) markers with representative H&E staining in the testis (h, i). The values are mean ± SEM; ∗ denotes a significant difference, *p* < 0.05; ∗∗ denotes a significant difference, *p* < 0.01.

As shown in Figure 8, all applied protocols in this study significantly affected oxidative status in rats' testicular tissue by means of lipid peroxidation (TBARS, Figure 8(a), *F* = 10.130), as well as both the enzymatic (SOD and CAT, Figures 8(b) and 8(c), *F* = 12.157 and 8.045, respectively) and nonenzymatic antioxidant mechanisms (GSH, Figure 8(d), *F* = 7.512). HA and ACP administration increased TBARS values when compared to the control (*p* < 0.01) and even when compared to the TCP group (*p* < 0.01) for ACP-treated animals. While antioxidant supplementation, when applied simultaneously with HA, resulted in a significant decline of lipid peroxidation compared to the HA group (*p* < 0.01) and reversed TBARS values to the control levels, FU extract did not significantly affect the ACP-induced increase in TBARS, and the values remained significantly above the control (*p* < 0.01). Interestingly, CAT activity in the testis was significantly reduced only in the ACP group when compared to the control group (*p* < 0.01), while the much more profound decline in SOD activity was observed in both the HA and ACP groups (*p* < 0.01). The prooxidant effect of ACP was even significant when compared to that of the TCP group (*p* < 0.01) and remained below the control values after simultaneous FU extract administration (*p* < 0.01). The ACP prooxidative action was also observed by means of GSH concentration in testicular tissue. Prolonged intake of ACP significantly declined GSH when compared to the control and TCP groups (*p* < 0.01). When FU extract was administered along with ACP, GSH levels remained below the control values (*p* < 0.05). The equilibrium in the relative gene expression of pro- and antiapoptotic factors was also significantly shifted toward the apoptosis by means of the relative Bax (Figure 8(e), *F* = 31.611) and Bcl-2 expression (Figure 8(f), *F* = 17.877). Bax relative gene expression in testicular tissue was significantly enhanced by HA and ACP administration when compared to the control (*p* < 0.01), as well as to the TCP group (*p* < 0.05 for HA, and *p* < 0.01 for ACP). Also, Bax observed in the ACP group was significantly above the values in the HA group (*p* < 0.05). Although the antioxidant supplementation significantly reduced Bax relative gene expression when compared to the group where CaPs were applied solely (*p* < 0.05 for HA, and *p* < 0.01 for ACP), Bax was significantly above the control values in the ACP+FU group (*p* < 0.01). At the same time, Bcl-2 expression in testicular tissue was significantly declined by all three applied nanosized CaPs when compared to the control (*p* < 0.01), with the most prominent response to ACP (*p* < 0.01 when compared to TCP). The antioxidant supplementation with FU extract failed to significantly improve the antiapoptotic capacity since the values were kept persistently below the control values (*p* < 0.05 for HA, and *p* < 0.01 for TCP and ACP). The proapoptotic action of nanosized CaPs in the male rat testis was confirmed by significant alterations in the Bax/Bcl-2 ratio (Figure 8(g), *F* = 62.745). However, the most significant changes were observed in the ACP group where the Bax/Bcl-2 ratio was significantly above the control, as well as the HA and TCP groups (*p* < 0.01). Although FU extract, when applied along with ACP, induced a significant decline in the Bax/Bcl-2 ratio (*p* < 0.01), the values remained above the control (*p* < 0.01). Pathohistological analysis of testicular tissue demonstrated notable changes in tissue architecture in ACP-treated rats. A remarkably lower number of testicular interstitial cells were present. Also, in interstitial spaces of ACP-treated rats, cells with condensed chromatin with pyknotic nuclei were present. In this experimental group, the seminiferous tubule structure was also notably altered with a diminished number of cells in the germinal epithelium (Figures 8(h) and 8(i)).

Summarizing the results obtained in individual organs, we presented (Table 1) the comparison of the observed effects for each experimental protocol.

## 4. Discussion

Although calcium phosphates may be considered materials for the future in various biomedical applications, potential health risks related to their usage are often underestimated, especially since the exposure to nanoparticles is continual and usually occurs without explicit consent. Even more, there is an evident imbalance in public views between the promotion of the benefits for new nanomaterials, including nano-CaPs, and the caution considering their side effects (Table 1). Therefore, it seems necessary to alert the public about the potential risk factors associated with their medical applications. In that sense, in order to allow a better insight into the nanoparticles' side effects, the knowledge in this field should be presented more systematically. First of all, investigations in this medical problem should be standardized by means of the particle size and shape, surface composition, and release of biologically active species [34]. Finally, the administration route should be considered one of the most important aspects of experimental design.

Although chronic nano-CaP administration resulted in negligible alterations in renal function indicators in peripheral blood, the analysis of oxidative stress indicators in renal tissue revealed that nano-CaP protocols produced significant worsening in oxidative stress markers. Prooxidative action of all three applied nano-CaPs was manifested by both increased ROS production (augmentation of the index of lipid peroxidation, Figure 6(a)) and diminished antioxidant capacity. Interestingly, both the enzymatic (SOD and CAT activities, Figures 6(b) and 6(c), respectively) and nonenzymatic (GSH, Figure 6(d)) antioxidant mechanisms were impaired, but it should be noticed that GSH levels were minimized only following prolonged HA administration. Furthermore, it seems obvious that simultaneous antioxidant supplementation with FU extract prevented the prooxidative action of the applied nano-CaPs within all estimated oxidative stress indicators in renal tissue. Thus, FU extract not only successfully diminished nano-CaP-induced ROS overproduction (except in the ACP group) but also restored the antioxidant capacity by means of increased enzymatic antioxidant activity, as well as by reversing GSH levels to the control values. Due to the lack of data for the action of other nano-CaPs on renal function *in vivo*, we can only compare our results with the most comprehensively evaluated nephrotoxic effects of HA. Indeed, our results are in accordance with the results for HA-induced nephrotoxicity obtained under various experimental conditions. Mosa and coworkers also reported prooxidative action of HA in renal tissue following chronic administration in male rats [16], which was manifested by the increased ROS production and marked decline in all antioxidant defense mechanisms, including total antioxidant capacity. The proposed mechanism of HA prooxidative action was based on previous findings that nanoparticles inside the cell promote imbalance between oxidants and antioxidants with consequent intracellular oxidative stress [35]. The intracellular prooxidative action of nanoparticles may be manifested by the affection in various intracellular structures based on the modification of lipids, proteins, and nucleic acids [36]. Although with no specific data for renal tissue, the observed proapoptotic action of nano-CaPs following the protocols applied in this study can be only compared to the previously reported general prooxidant pathways described for nano-HA *in vitro*. Xu and collaborators observed the dose-dependent prooxidative action in C6 cells as an acute response to nano-HA particles [36]. A similar prooxidant effect of nano-HA was confirmed on osteoblastic MC3T3-E1 cells, particularly affecting mitochondrial pathways [13]. These investigators, although with different doses and time exposure, reported both increased ROS production and diminished antioxidant capacity following HA administration.

The impact of the applied nano-CaPs on apoptotic markers was significant only in the ACP group (Figures 6(e)–6(g)). Interestingly, the proapoptotic action of ACP was confirmed not only when compared to the control group but also in comparison to the other two applied nano-CaPs and persisted even after antioxidant supplementation with FU extract. Again, with no specific data for the impact of nano-CaPs on apoptotic indicators in renal tissue, we can only state that our results are in line with previous reports for proapoptotic action of nano-CaPs obtained in *in vitro* studies. HA nanoparticle administration resulted in potentiating apoptosis in osteoblasts and macrophages via the augmentation of p53 expression and caspase family activity and the simultaneous downregulation of Bcl-2 [37, 38]. At the same time, it has been reported that ACP nanoparticles induced apoptosis of leukemia cells by the selective effect in the G1 phase [39].

The most prominent prooxidative and apoptotic actions, as observed in the ACP group, were accompanied by the morphological alterations in renal tissue, predominantly manifested by vacuolation of the proximal convoluted tubule epithelium and glomerular cells (Figures 6(h) and 6(i)). It is not surprising that kidney tissue undergoes significant alterations following potentially toxic substances due to the high flow rate of blood, which in turn delivers elevated concentrations of nano-CaPs to the kidney. Moreover, the proximal tubule epithelium is more vulnerable to nephrotoxicity, as confirmed in this study. This specific local affection can be attributed to the fact that these cells express various transporters, which enable active intake and intracellular accumulation of toxic compounds [40].

Under the standardized conditions that allow the comparison of individual nano-CaP effects on renal function, as performed in this study, it seems that the nephrotoxicity by means of all estimated levels was the most prominent in the ACP-treated group, with the less harmful effect observed in the HA group, and especially in TCP-treated rats. However, it should also be taken into account that the increased phosphate load itself may significantly affect kidney morphology and function [41]. Therefore, it is not surprising that hypercalcemia was confirmed only following the treatment with ACP. The observed serum calcium level elevation achieved with prolonged ACP oral intake was significant not only when compared to the control but also when compared to the TCP group. However, simultaneous administration of FU extract was sufficient to attenuate the ACP-induced calcemia rise.

As expected, we are not able to compare our results for the impact of antioxidant supplementation with FU extract to other reports that used the same antioxidant-rich natural compound. Therefore, we can confirm that the protective role of antioxidants, as observed in this study, is in accordance with the previously reported beneficial effect of curcumin and chitosan on HA-induced nephrotoxicity mechanisms by means of their antioxidative and antiapoptotic actions, accompanied by the restoration of tissue architecture [16].

None of the evaluated liver enzymes was affected by the prolonged oral intake of nano-CaPs in doses applied in this study (Figures 4(b)–4(d), respectively), but LDH levels were significantly above the control values following prolonged ACP administration. The serum LDH observed in the ACP group was also significantly higher when compared to that in TCP-treated animals. However, this effect of ACP was prevented by simultaneous administration of FU extract (Figure 4(a)). On the other hand, the indicators of liver metabolic functions were significantly influenced by the applied protocols. Interestingly, while neither HA nor TCP altered serum levels, ACP administration increased serum levels of triglycerides, LDL, and total cholesterol, as well as the cholesterol ratio (Figures 3(a)–3(e)). Those manifestations of dyslipidemia were abolished by antioxidant supplementation with FU extract. The results obtained in this study are in accordance with previous findings of Chen and colleagues [42], in terms of increased LDH and unaltered ALP levels following nano-HA administration. A similar increase in serum LDH levels following HA nanoparticle application was also achieved in a dose-dependent manner in an *in vitro* experimental model [17]. In contrast to the previously mentioned investigation, treatments with nano-CaPs applied in this study did not increase serum ALT and AST levels. Those discrepancies could be attributed to differences in experimental design, including the applied dose (three times above ours), the exposure duration, and the route of administration. This can also be considered an indirect confirmation that liver injury following nano-CaP administration in this study was not as severe as in the investigation conducted by Chen et al. Indeed, a recent study by Paraš and coworkers [43] also showed no significant alterations in AST and ALT levels following chronic administration of nano-HA (120 days). The lipid profile alterations observed in our study are also in accordance with the reported increase in serum total cholesterol and LDL levels following nano-HA administration [42].

The evaluation of oxidative stress markers in hepatic tissue revealed a significant impact of the applied protocols. ROS production was significantly enhanced by HA and ACP, with no significant effect of TCP (Figure 7(a)). This manifestation of prooxidative action was successfully abolished by FU extract in the HA group but remained above the control values in ACP-treated animals. At the same time, the enzymatic antioxidant defense was markedly reduced by HA and ACP, again with no significant impact of TCP (Figures 7(b) and 7(c), respectively). Interestingly, the decline in enzymatic antioxidant activity in the HA and ACP groups was significantly augmented even when compared to that in the TCP group and remained lower after simultaneous antioxidant supplementation. Furthermore, GSH levels in hepatic tissue samples were reduced only in ACP-treated rats, but this was prevented by FU extract. Our results correspond to the data obtained in the rat liver cell model [17], where HA nanoparticles induced the dose-dependent augmentation of total oxidative stress and the simultaneous decline in total antioxidant capacity. Also, a single dose of 50 mg/kg nano-HA resulted in prooxidative action in rat liver samples [42]. Like in our study, even an acute response to HA nanoparticles was manifested by increased lipid peroxidation, decreased SOD activity, and diminished GSH content. Due to the lack of literature data for other nano-CaPs, we can only compare our results with other nanometallic compounds and assume that the prooxidative action, as observed in this study, might be mediated via the JNK/p53 and NF-*κ*B pathways [44].

The proapoptotic effect of the applied nano-CaPs was expressed by means of both the increase of the relative proapoptotic gene expression (Figure 7(e)) and the decline in the relative antiapoptotic gene expression (Figure 7(f)) and confirmed by their ratio (Figure 7(g)). Unlike the oxidative stress, apoptotic markers were significantly affected by all three applied nano-CaPs, but this effect was prevented by simultaneous FU extract administration except in ACP-treated rats. Structural alterations accompanied by prooxidative and apoptotic actions of nano-CaPs in liver tissue samples were manifested predominantly through lipid accumulation in the form of intralobular microvesicular steatosis. Not surprisingly, the morphological changes were the most prominent in the ACP group (Figures 7(h) and 7(i)). Again, although with a different experimental design, it is obvious that our results are in line with the previously reported proapoptotic action of nano-HA [42]. However, structural changes described in that investigation do not correspond to ours. Namely, in contrast to Chen et al.'s report that potentiates inflammatory response in the liver following HA administration, our experimental protocols resulted in microvesicular steatosis. The observed differences could be explained by the fact that the acute response to the high dose of nano-HA could trigger an immediate hepatotoxic effect manifested by inflammatory cell infiltration, while chronic treatment with the lower dose of nano-HA predominantly caused the steatotic effect.

The obvious beneficial role of antioxidant supplementation with FU extract on nano-CaP-induced hepatotoxicity cannot be compared to similar reports. However, although already known for its protective role by means of antioxidant and anti-inflammatory effects [24], the confirmation of benefits of using FU extract following nano-CaP administration could be found only in certain brain regions [11].

A significant decline in serum testosterone levels (for approximately 50%) was observed in the HA and ACP groups, with no significant impact of TCP (Figure 5(a)). Serum testosterone levels decrease induced by nano-HA, and ACP was successfully attenuated by FU extract administration. All applied nano-CaPs diminished LH levels in sera, but unlike testosterone, the decline in serum LH persisted in all experimental groups even with simultaneous antioxidant supplementation (Figure 5(b)). The decline of testosterone, as observed in this study, following nano-CaPs is in accordance with the recently reported effect of chronic nano-HA on serum testosterone levels, while the lowering of LH is not in line with the results of a similar investigation [18]. Therefore, we must notice that there was a significant difference in experimental design in the mentioned study in which the pretreatment was 50% longer, and the daily dose was more than 15-fold higher. However, the principal mechanism of sex hormone level alterations may be found in the significant reduction of interstitial cells in testes that are the main source of testosterone. Furthermore, this mechanism is probably responsible for the observed changes in the germinal epithelium.

The prooxidative action of nano-CaPs in testicular tissue was confirmed by various aspects. The increased ROS production and decline in SOD activity were observed in HA- and ACP-treated animals (Figures 8(a) and 8(c)), while only nano-ACP administration reduced CAT activity and GSH levels (Figures 8(b) and 8(d)). The most pronounced prooxidative action of ACP remained persistent after simultaneous administration of FU extract. It is worth noticing that TCP administration induced no significant prooxidative response. The same response to nano-HA was observed in the study that evaluated the reproductive toxicity [18]. Due to the lack of other literature sources for nano-CaPs, we can only compare our results with previously reported oxidative damage in other species induced by different metallic nanoparticles [45].

While all three administered nano-CaPs induced a decline in Bcl-2 relative gene expression (Figure 8(f)), only nano-HA and ACP increased Bax relative gene expression in testicular tissue. On the other hand, FU extract application significantly lowered Bax but did not affect Bcl-2 alterations induced by nano-CaPs. Again, the proapoptotic potential, expressed as the Bax/Bcl-2 ratio, reached the highest levels in ACP-treated animals. The results obtained in this study for the proapoptotic action may be supported by the previously reported effect of nano-HA that involves the increase in p53 and TNF-*α* [18].

In testicular tissue, we observed significant alterations in tissue architecture following ACP treatment. The most prominent difference, when compared to the control group, was a reduction in the number of testicular interstitial cells. This was accompanied by the diminished number and altered structure of the germinal epithelial cells (Figures 8(h) and 8(i)). Our results are in line with the reduction of Leydig cells as the consequence of nano-HA treatment [18], as well as with the report that nano-Ag particles reduced the number of germline stem cells in testes [45].

The beneficial response to simultaneous administration of FU extract manifested by counteracting nano-CaP-induced gonadal dysfunction was confirmed at different estimated levels: sex hormone serum levels, oxidative and proapoptotic indicators, and structural changes in testicles. However, there is no literature data for this specific action of FU, so we can only comment that our results are in accordance with the previously reported protective role of other antioxidants (such as curcumin and chitosan) on nano-CaP-induced gonadotoxicity [18].

## 5. Conclusions

The results of our study clearly demonstrate that chronic intake of nano-CaPs may be accompanied by serious systemic and organ-specific adverse effects. Therefore, we suggest that further investigations for potential medical application of novel materials for tissue engineering should include at least a basic systematic estimation of side effects, in order to achieve the biosafety of new therapeutic compounds. Yet, at least some aspects of observed toxicities may be prevented by safe and reliable supplementation with antioxidants.

## Figures and Tables

**Table 1:** Summary of the organ-specific results.

		Effect (organ)					
Treatment		Oxidative damage		Apoptosis		Structural alterations	
		*Kidney*					
HA	HA+FU	↑↑↑	↓↓	n.c.	n.c.	n.c.	n.c.
TCP	TCP+FU	↑↑↑	↓↓	n.c.	n.c.	n.c.	n.c.
ACP	ACP+FU	↑↑↑	↓↓	↑↑	↓↓	↑	↓
		*Liver*					
HA	HA+FU	↑↑	↓↓	↑↑	↓	n.c.	n.c.
TCP	TCP+FU	↑	↓	↑	↓	n.c.	n.c.
ACP	ACP+FU	↑↑↑	↓	↑↑↑	↓↓	↑	↓
		*Testis*					
HA	HA+FU	↑↑	↓↓↓	↑↑	n.c.	n.c.	n.c.
TCP	TCP+FU	n.c.	n.c.	↑	n.c.	n.c.	n.c.
ACP	ACP+FU	↑↑↑	↓	↑↑↑	↓↓	↑↑	↓

The arrows represent the description of alterations in the evaluated parameters. n.c. = no change.

## Data Availability

All data is available upon request.
